# Antibacterial activity of plasma from crocodile (*Crocodylus siamensis*) against pathogenic bacteria

**DOI:** 10.1186/1476-0711-11-22

**Published:** 2012-07-30

**Authors:** Jintana Kommanee, Sutthidech Preecharram, Sakda Daduang, Yosapong Temsiripong, Apisak Dhiravisit, Yuzo Yamada, Sompong Thammasirirak

**Affiliations:** 1 Department of Biochemistry, Faculty of Science, Protein Proteomic Research Group, Khon Kaen University, Khon Kaen, 40002, Thailand; 2 Department of Microbiology, Faculty of liberal arts and science, Kasetsart University Kamphaeng saen Campus, Nakhon Pathom, 73140, Thailand; 3 Sriracha Moda Farm, Chon Buri, 20110, Thailand; 4 Faculty of Humanities and Social Sciences, Khon Kaen University, Khon Kaen, Thailand, 40002; 5 BIOTEC Culture Collection (BCC), National Center for Genetic Engineering and Biotechnology (BIOTEC), Pathumthani, 12120, Thailand; 6 JICA Senior Overseas Volunteer, Japan International Cooperation Agency (JICA), Shibuya-ku, Tokyo, 151-8558, Japan; 7 Shizuoka University, Suruga-ku, Shizuoka, 422-8529, Japan

**Keywords:** Crocodile (*Crocodylus siamensis*), Antibacterial activity, Pathogenic bacteria, Cytotoxicity

## Abstract

**Background:**

The Siamese crocodile (*Crocodylus siamensis*) is a critically endangered species of freshwater crocodiles. Crocodilians live with opportunistic bacterial infection but normally suffer no adverse effects. They are not totally immune to microbial infection, but their resistance thereto is remarkably effective. In this study, crude and purified plasma extracted from the Siamese crocodile were examined for antibacterial activity against clinically isolated, human pathogenic bacterial strains and the related reference strains.

**Methods:**

Crude plasma was prepared from whole blood of the Siamese crocodile by differential sedimentation. The crude plasma was examined for antibacterial activity by the liquid growth inhibition assay. The scanning electron microscopy was performed to confirm the effect of crude crocodile plasma on the cells of *Salmonella typhi* ATCC 11778. Effect of crude crocodile plasma on cell viability was tested by MTT assay. In addition, the plasma was purified by anion exchange column chromatography with DEAE-Toyopearl 650 M and the purified plasma was tested for antibacterial activity.

**Results:**

Crude plasma was prepared from whole blood of the Siamese crocodile and exhibited substantial antibacterial activities of more than 40% growth inhibition against the six reference strains of *Staphylococcus aureus*, *Salmonella typhi*, *Escherichia coli*, *Vibrio cholerae*, *Pseudomonas aeruginosa*, and *Staphylococcus epidermidis*, and the four clinical isolates of *Staphylococcus epidermidis*, *Pseudomonas aeruginosa*, *Salmonella typhi*, and *Vibrio cholerae*. Especially, more than 80% growth inhibition was found in the reference strains of *Salmonella typhi*, *Vibrio cholerae*, and *Staphylococcus epidermidis* and in the clinical isolates of *Salmonella typhi* and *Vibrio cholerae*. The effect of the crude plasma on bacterial cells of *Salmonella typhi*, a certain antibacterial material probably penetrates progressively into the cytoplasmic space, perturbing and damaging bacterial membranes. The effect of the crude plasma was not toxic by the yellow tetrazolium bromide (MTT) assay using a macrophage-like cell, RAW 264.7. The pooled four fractions, designated as fractions D1-D4, were obtained by column chromatography, and only fraction D1 showed growth inhibition in the reference strains and the clinical, human pathogenic isolates.

**Conclusions:**

The crude and purified plasma from the Siamese crocodile significantly showed antibacterial activity against pathogenic bacteria and reference strains by damage cell membrane of target bacterial cells. From the MTT assay, the Siamese crocodile plasma was not cytotoxic to the cells.

## Background

The powerful antibiotic was first discovered in 1940s [1], many of which originate from natural sources. Meanwhile, microbes resistant to these antibiotics continuously emerge over time and spread all over the world. This situation motivated scientists to search for new naturally occurring bacterial agents that may be potential antibiotics. Reptiles including alligators and crocodiles were subjected to an exploration of the antibiotic properties from their body.

Bacteria and fungi coexist with other living organisms. Despite such an ecological phenomenon, pathogenic microbial invasion into hosts does not normally take place, as the host has the defense mechanisms that counteract infection. One of such systems is based on non-specific immunity, which comprises a wide variety of peptides [2] and factors [3] with potent antimicrobial properties.

A kind of crocodiles (*Crocodylus siamensis*), the Siamese crocodile is a critically endangered species of freshwater crocodiles, originally distributed in most of South East Asia. Crocodilians live with opportunistic bacterial infection but normally suffer no adverse effects. They are not totally immune to microbial infection, but their resistance thereto is remarkably effective. The immune system of crocodilians has not been well characterized, but there are several reports that describe the antimicrobial efficacy of alligator serum towards bacteria, viruses, and amoeba [4-6]. Merchant *et al.*[5] recently proposed that the complement systems of alligators are effective in killing bacteria. More recently, leukocyte extract of the American alligator (*Alligator mississippiensis*) showed a broad spectrum of antibiotic properties on bacteria, fungi, and viruses [7,8]. Effects of bacterial lipopolysaccharide on peripheral leukocytes were also investigated in the American alligator [9].

In the present study, we have examined the crude and purified plasma obtained from the Siamese crocodile for the antibacterial activity against clinically isolated, human pathogenic bacterial strains and the related reference strains, which will be of great importance in medical industry.

## Materials and methods

### Plasma samples

Crocodiles (*Crocodylus siamensis*) were captured and housed at the local Sriracha Moda Farm, Chon Buri, Thailand. The crocodiles (age ranging from 1–3 years) were housed in a single tank, and treated with electric shock. Blood samples were collected from the dorsal vein using a heparinized 38 mm long 18 gauge needle and a 60 ml syringe and transferred to heparinized vacuum tubes. The crocodile blood in heparinized vacuum tubes was kept at 4°C for overnight and then centrifuged 800 *g* to obtain crude plasma, which was kept at −70°C until used [1-3,7,10-16].

### Bacterial strains

*Staphylococcus aureus* ATCC 25923, *Salmonella typhi* ATCC 11778, *Escherichia coli* O157:H7, *Vibrio cholerae* non01, *Pseudomonas aeruginosa* ATCC 27853, *Staphylococcus epidermidis* ATCC 12228, *Staphylococcus epidermidis* clinical isolate 1, *Pseudomonas aeruginosa* clinical isolate 1, *Salmonella typhi* clinical isolate 1, and *Vibrio cholerae* clinical isolate 1 were maintained in nutrient agar slants at 4°C.

### Purification of plasma by DEAE

After blood centrifugation for plasma collection, crude plasma 200 ml was diluted with 18 ml of 25 mM sodium acetate buffer, pH 5.0 and stirred for 10 min at room temperature. The homogenate was centrifuged at 12000 *g* for 15 min. The supernatant was collected, stirred, and incubated with CM-Toyopearl 650 M cation exchange resin. The incubated resin was packed on column and washed with 2 cv of 25 mM acetate buffer, pH 5.0. The un-absorbed protein fraction including washings was adjusted to pH 8.1 and then loaded onto the DEAE-Toyopearl 650 M anion exchange resin equilibrated with 25 mM Tris–HCl, pH 8.1. The absorbed fraction was eluted with stepwise NaCl concentration of 0.1, 0.2, 0.3, 0.4, and 0.5 M at flow rate of 1.0 ml/min. The eluted fraction was collected and monitored at A280 nm for determining the peak of protein.

### Assay of antibacterial activity

The antimicrobial activity of the crude plasma preparation was evaluated against the four pathogens and six reference strains. The antibacterial activity was measured by the disc diffusion method [17] with modifications. The inoculation of each bacterium was done by placing inoculum 10^7^ CFU/ml on Mueller-Hinton agar (Scharlau, Spain). After drying the agar for 3 to 5 min, 6 mm discs were applied to each plate, and the DEAE eluate fractions (25 μl; concentration, 1 mg/ml) was pipetted onto each disc. The agar plates were incubated at 37°C for 18 h. Clear zone diameter was measured with a ruler at the back of the plate. Deionized distilled water (DDW) and streptomycin 10 μg on discs were used respectively as negative and positive control assays.

The DEAE eluate fractions were assayed for antibacterial activity against the ten test strains mentioned above by a liquid growth inhibition assay [18]. DDW and streptomycin 3 μg were used respectively as negative and positive controls. The antimicrobial activity tests were done in triplicate to obtain valid statistical evaluation of the results, which were expressed as mean ± S.D. The percentage of the growth inhibition was calculated with absorbance values using the following equation, CI (%) = (A - B)/A × 100, where CI is the percentage inhibition index, A is the value observed at A550 nm of bacteria for negative control, and B is the value observed at A550 nm of bacteria for a plasma eluate or streptomycin.

### Scanning electron microscopy

The scanning electron microscopy (SEM) was performed according to Lau *et al.*[19] with slight modifications. *Salmonella typhi* ATCC 11778 was grown in nutrient broth and harvested at the logarithmic phase of growth by centrifugation at 3,000 *g* for 5 min. The bacterial cells were then washed twice with phosphate buffered saline (PBS), pH 7.0 and re-suspended to the final concentration of 10^6^ CFU/ml. Aliquots of suspension of *Salmonella typhi* ATCC 11778 (100 μl) were individually incubated with the crude plasma (300 μg/50 μl) at 37°C for 2 h. The incubated bacterium 150 μl was fixed with equal volumes of 2.5% glutaraldehyde (Sigma, USA) in 0.1 M phosphate buffer, pH 7.2 for 2 h. The fixed cells were carefully pipetted and settled onto a 0.2 μm polycarbonate membrane filter (Whatman, Germany) for five min and then washed twice with PBS, pH 7.0. The fixed material was dehydrated by rinsing for 15 min repeatedly with ethanol solutions, of which the concentration was elevated stepwise from 30%, 50%, 70%, 90%, and finally 100% ethanol. The dehydrated material in the absolute ethanol was dried in a critical point drier (Critical point drier, Balzers model CPD 020) with carbon dioxide as the drying agent. The dry material was coated by sputter coater (Sputter Coater, Balzers model SCD 040) with gold palladium and examined by a scanning electron microscope (JEOL, model JSM-5410LV). The negative control was performed in a similar manner except that the bacterial cells were incubated with PBS, pH 7.0 instead of the crude crocodile plasma.

### Cell line

The mouse macrophage cell line, RAW 264.7 was obtained from the European Collection of Cell Cultures (ECACC) and cultured in RPMI medium supplemented with 10% heat-inactivated fetal bovine serum (FBS), penicillin 100 units/ml, streptomycin 100 μg/ml, and amphotericin B 25 μg/ml were maintained at 37°C in a 5% CO_2_ humidified atmosphere (CO_2_ incubator, Heal Force). The cells were treated with the crude plasma at different concentrations during 1 h for the indicated period.

### MTT assay for cell viability

Cells were seeded at a density of 104 cells/well in 96-well plates overnight, followed by the treatment with different concentrations of the crude plasma, 62.5, 125, 250, 500, and 1,000 μg/ml. The cell viability of RAW 264.7 was measured after 24 h exposure to the test plasma by colorimetric assay, based on the ability of mitochondria in viable cells to reduce MTT [3-(4,5-Dimethylthiazol-2-yl)-2,5-diphenyltetrazolium bromide]. An aliquot of MTT solution 0.5 mg/ml was added to each well, and after 30 min incubation at 37°C, the medium was discarded, and the formazan blue formed in the cells was dissolved in dimethyl sulfoxide (DMSO). Absorbance at 570 nm was determined with a microplate reader (Bio-Rad, Model 680, USA). The absorbance of the formazan formed in non-treated cells was taken as 100% viability.

### Statistical analysis

Data points in all experiments were performed in triplicate to obtain valid statistical evaluation of the results. Each sample CFUs/ml was calculated by multiplying the number of colonies counted by the dilution factor. All results represented the means ± S.E.M for at three determinations.

## Results

### Antibacterial activity of crude crocodile plasma

**Figure 1:**
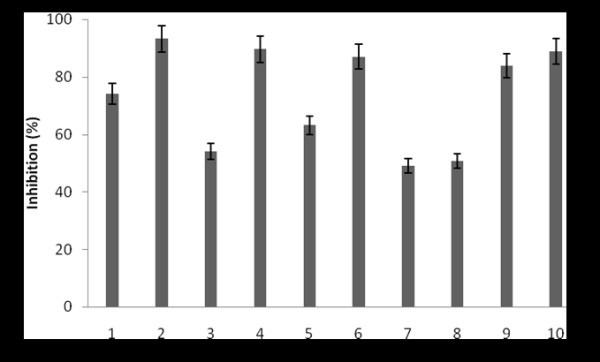
**The antibacterial effect of the crude Siamese crocodile plasma on pathogenic bacteria.** The final protein concentration of the crude plasma examined was 50 μg/ml. 1, *Staphylococcus aureus* ATCC 25923; 2, *Salmonella typhi* ATCC 11778; 3, *Esccherichia coli* O157:H7; 4, *Vibrio cholerae* non01; 5, *Pseudomonas aeruginosa* ATCC 27853; 6, *Streprococcus epidermidis* ATCC 12228; 7, *Streprococcus epidermidis* clinical isolate 1; 8, *Pseudomonas aeruginosa* clinical isolate 1; 9, *Salmonella typhi* clinical isolate 1; 10, *Vibrio cholerae* clinical isolate 1. Bars represent the mean and the standard deviations performed in triplicate.

The undiluted crude Siamese crocodile plasma containing 114 mg protein/ml determined by the Bradford assay (Bradford, 1976) was examined for antibacterial activity by the liquid growth inhibition assay. The crocodile plasma was effective as an antibacterial substance against the following six reference strains and four pathogenic isolates significantly: *Staphylococcus aureus* ATCC 25923, *Salmonella typhi* ATCC 11778, *Escherichia coli* O157:H7, *Vibrio cholerae* non01, *Pseudomonas aeruginosa* ATCC 27853, *Streptococcus epidermidis* ATCC 12228, *Streptococcus epidermidis* clinical isolates 1, *Pseudomonas aeruginosa* clinical isolate 1, *Salmonella typhi* clinical isolate 1, and *Vibrio cholerae* clinical isolate 1. The inhibition effects on the bacterial strains were mostly over 40% (Figure 1). Especially, the crocodile plasma showed high inhibition effects more than 80% on *Salmonella typhi* ATCC 11778, *Vibrio cholerae* non01, *Streptococcus epidermidis* ATCC 12228, *Pseudomonas aeruginosa* clinical isolates 1, and *Salmonella typhi* clinical isolates 1. The experimental data obtained indicated that the crude Siamese crocodile plasma had antibacterial properties and bactericidal actions to some bacterial strains both gram-positive and gram-negative.

### Antibacterial action of crocodile plasma examined by scanning electron microscopy

**Figure 2:**
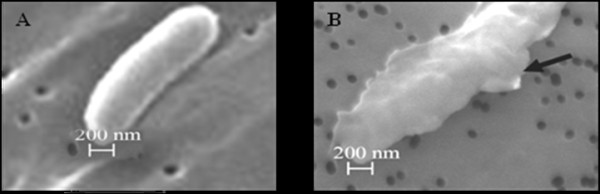
**The antibacterial effect of the crude Siamese crocodile plasma on cells of*Salmonella typhi*ATCC 11778.** The cells were incubated with the crude plasma for 120 min and observed by scanning electron microscopy. **a**, without incubation; **b**, with incubation.

The effect of the crude crocodile plasma on the cell of *Salmonella typhi* ATCC 11778 tested as a reference strain was examined by SEM. As shown in Figure 2a, the untreated cells had smooth, intact cell surfaces. In contrast, crude plasma-induced breakage and roughness was shown in the cell surfaces after a 120 min treatment (Figure 2b). The results obtained indicated that the cell surface including bacterial cell walls and/or cell membranes was an important target for action by the crocodile plasma.

### Effect of crude crocodile plasma on cell viability

**Figure 3:**
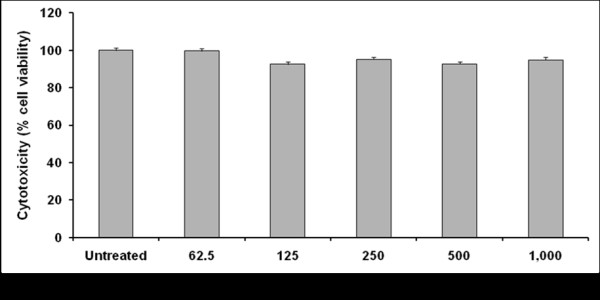
**The effect of the crude Siamese crocodile plasma on a macrophage-like cell, RAW 264.7 determined by the MTT assay.** The final protein concentration of the plasma tested was 1000 μg/ml. The cell viability without crude plasma was taken as 100%, where n = 8 (mean ± S.E.M.).

To assess the cytotoxicity of the crocodile plasma administered to the cells, macrophages RAW 264.7 was used. Although the MTT assay is an indirect measurement of cell density or the number of living cells attached to the culture plate by formation of colored formazan crystals, the crocodile plasma exhibited almost similar viability of more than 96% to that of non-treated cells, which was taken as 100% viability (Figure 3). The results obtained indicated that the crocodile plasma was not cytotoxic to the cells.

### Antibacterial activity of partially purified crocodile plasma

**Figure 4:**
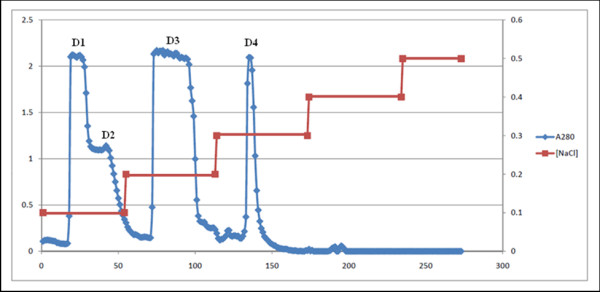
**The DEAE chromatography of the crude Siamese crocodile plasma.** The chromatography was made on a DEAE-Toyopearl 650 M column. Fraction D1 contained fraction tube numbers 19–24, fraction D2 contained fraction tube numbers 27–72, fraction D3 contained fraction tube numbers 73–96, and fraction D4 contained fraction tube numbers 133–142. ●, absorbance at 280 nm; ■, concentration of NaCl.

The Siamese crocodile plasma was separated into four pooled fractions by anion exchange column chromatography with DEAE-Toyopearl 650 M, designated as fractions D1, D2, D3, and D4 (Figure 4). Fractions D1 and D2 were eluted at 0.1 M NaCl concentration, whereas fractions D3 and D4 were eluted respectively at 0.2 and 0.3 M NaCl. All the fractions were assayed for antimicrobial activity.

In the disc diffusion assay that was made as a qualitative analysis, some of the four fractions exhibited antibacterial activities against all the tested ten pathogenic bacteria (Table 1). The most active fractions were found in fraction D1 in all the ten test strains. The activity of the other three fractions, D2, D3, and D4 was, however, calculated to be 50% or less of that of fraction D1.

**Table 1:** The antibacterial activity of the DEAE eluate fractions determined by the disc diffusion assay

**Bacterial strain**	Inhibition zone (mm) of fraction*			
	**D1**	**D2**	**D3**	**D4**
*S. aureus* ATCC 25923	9.6	2.4	2.5	-
*S. typhi* ATCC 11778	12.4	-	-	-
*E. coli* O157:H7	5.5	2.2	2.8	-
*V. cholerae* non01	10.8	3.1	-	-
*P. aeruginosa* ATCC 27853	6.7	2.5	1.8	-
*S. epidermidis* ATCC 12228	14.2	1.8	-	-
*S. epidermidis* clinical isolate 1	12.5	2.4	2.5	-
*P. aeruginosa* clinical isolate 1	6.5	1.8	1.5	-
*S. typhi* clinical isolate 1	11.8	2.8	2.2	1.3
*V. cholerae* clinical isolate 1	9.7	2.6	2.0	1.4

For the assay condition, see the text. -, no growth inhibition.

*The values not include diameter of paper disc.

**Figure 5:**
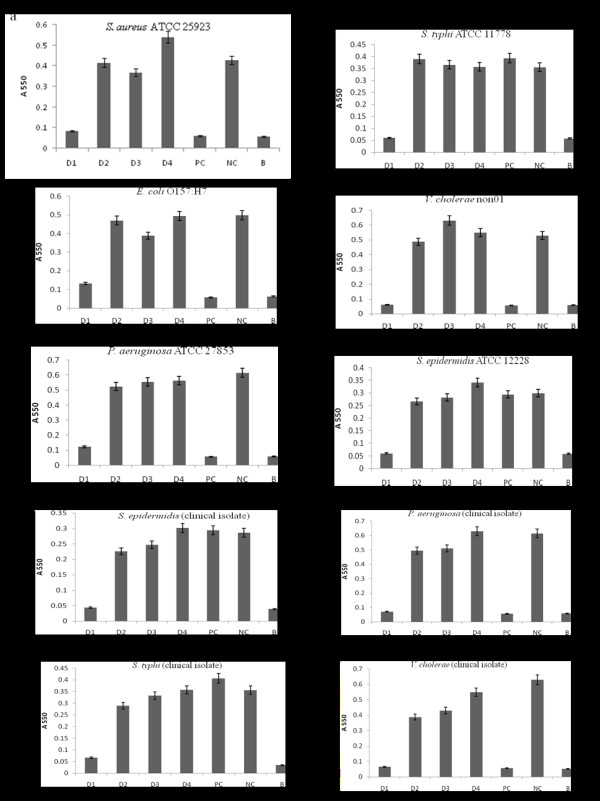
**The antibacterial activity of the DEAE eluate fractions determined by the liquid growth inhibition assay.** The growth inhibition test was made for 16 h with the final protein concentration 50 μg/ml of fraction D1, D2, D3, or D4. **a**, *Staphylococcus aureus* ATCC 25923; **b**, *Salmonella typhi* ATCC 11778; **c**, *Escherichia coli* O157:H7; **d**, *Vibrio cholerae* non01; **e**, *Pseudomonas aeruginosa* ATCC 27853; **f**, *Streptococcus epidermidis* ATCC 12228; **g**, *Staphylococcus epidermidis* clinical isolate; **h**, *Pseudomonas aeruginosa* clinical isolate 1; **i**, *Salmonella typhi* clinical isolate 1; **j**, *Vibrio cholerae* clinical isolate 1; PC, with streptomycin 3 μg/ml in the final concentration; NC, without any of streptomycin and plasma fractions.

In the liquid growth inhibition assay that was made as a quantitative analysis, clear results were obtained (Figure 5). In only fraction D1, significantly inhibited the growth of all tested bacteria, which almost corresponded to that of streptomycin 3 μg/ml. Interesting is that the growth of both *Salmonella typhi* ATCC 11778 and clinical isolate 1 and *Streptococcus epidermidis* ATCC 1222 and clinical isolate 1 was not inhibited by streptomycin.

## Discussion

The antibacterial activity has been reported in the American alligator (*Alligator mississippiensis*), the saltwater crocodile (*Crocodylus porosus*), and the freshwater crocodile (*Crocodylus johnstoni*) [5,20]. However, only a few reports were found in one of the freshwater Siamese crocodiles [13]. In the present study, the Siamese crocodile plasma was effective as an antibacterial agent against the following six reference strains and the four pathogens both Gram-negative and Gram-positive species: *Staphylococcus aureus* ATCC 25923, *Salmonella typhi* ATCC 11778, *Ecsherichia coli. coli* O157:H7, *Vibrio cholerae* non01, *Pseudomonas aeruginosa* ATCC 27853, *Streptococcus epidermidis* ATCC 12228, *Streptococcus epidermidis* clinical isolate 1, *Pseudomonas aeruginosa* clinical isolate 1, *Salmonella typhi* clinical isolate 1, and *Vibrio cholerae* clinical isolate 1. All the bacterial species used in this study are known to be human pathogens. It is obvious that the Siamese crocodile plasma has a broad band of spectrum in antibacterial activities. The results demonstrated that the antibacterial activities were highly effective as an antibacterial agent against both Gram-positive and Gram-negative bacteria, consistent with the study in American alligator serum [6].

Antimicrobial peptides have been isolated from a broad variety of phylogenetically diverse organisms and separated into a variety of classes based on their chemical structures [21]. The classes of the peptides showed different kinds of mechanisms on antimicrobial action [13]. In the present study, the antibacterial mechanism of the crude Siamese crocodile plasma on the cell surface, including cell walls and membranes, of *Salmonella typhi* ATCC 11778, a reference strain was studied by SEM. The results obtained suggested that an antibacterial compound probably penetrates progressively into the cytoplasmic space, perturbing and damaging bacterial cell surface, as found in the previous report of Preecharram *et al*. [13], who isolated an antibacterial compound called crocosin from the *Crocodylus siamensis* plasma using RP-HPLC and found that the compound exhibited an antibacterial activity toward *Salmonella typhi* and *Staphylococcus aureus*. These results in the actions of the antimicrobial compounds correspond with the actions of the antimicrobial peptides such as bactenecin 5, bactenecin 7, poly-L-lysine, cecropin B, LL-37 PGYa, melittin, Hecate-1 and SMAP-29 [11,12,14,22-24]. Most antibacterial peptides found thus far, such as those from amphibian skin, magaingnin and dermaseptin are known to exert their antimicrobial activity by permeabilizing the membrane [11,23]. The antibacterial compound isolated by Preecharram *et al*. [13] has a similar mechanism on action to those of the antibacterial peptides reported [14,22] in disrupting the cell surface. However, all antimicrobial peptides have several factors, in common, which are able to interact with the negatively charged phospholipids in the membranes of microbes and are also amphipathic, so that the compounds interact with the interior hydrophobic portions of the membranes and form pores in the membranes [15].

However, there was no report about the cytotoxicity of the compound administered to cells of a macrophage-like cell, RAW 264.7. The present data is the first report. The MTT assay, to assess the cytotoxicity of the Siamese crocodile plasma administered to cells of macrophage RAW 264.7, is an assay of metabolic competence based on assessment of mitochondrial performance by colorimetrical measuring of the conversion of yellow tetrazolium bromide (MTT) to the purple formazan derivative by mitochondrial succinate dehydrogenase in viable cells [25]. In the present study, the wells incubated with the crocodile plasma showed light yellow color, whereas the wells incubated as negative controls showed deep purple. From the MTT assay mentioned above, the Siamese crocodile plasma was not cytotoxic to the cells.

## Conclusions

In summary, the crude and purified plasma from *Crocodylus siamensis* showed significantly antibacterial activity against pathogenic bacteria and reference strains by damage cell membrane of target bacterial. From the MTT assay, the Siamese crocodile plasma was not cytotoxic to the cells. On the basis of the results obtained above, the plasma extracted from *Crocodylus siamensis* was known to have a significant use as a clinical antimicrobial agent. So the subsequent studies are required to focus on the purification and characterization of the agent responsible for the antimicrobial activities including the anti-inflammatory activity.

## Competing interests

The authors declare that they no competing interests.

## Authors’ contributions

JK and SP carried out the study and wrote the manuscript. SD, AD, YT, YY and ST supervised the work and the manuscript. YY contributed to the manuscript corrections. All authors read and approved the final manuscript.
